# Supplementary material for: Impacts of environmental uncertainty on investment stocks perception under the holiday effect
Source: PLoS One. 2023 Aug 3;18(8):e0284745. doi: 10.1371/journal.pone.0284745 (PMC10399787; doi:10.1371/journal.pone.0284745)
Supplement: Attachment — Submitted filename: Response to Reviewer(2023.3.4).docx [file pone.0284745.s001.docx]

PONE-D-22-30888

The Impact of Environmental Uncertainty on The Perception of Investment Stocks Under the Holiday Effect

PLOS ONE

**Response to Reviewer**

1. Is the manuscript technically sound, and do the data support the conclusions?

The manuscript must describe a technically sound piece of scientific research with data that supports the conclusions. Experiments must have been conducted rigorously, with appropriate controls, replication, and sample sizes. The conclusions must be drawn appropriately based on the data presented.

Reviewer #1: Partly

Reviewer #2: Yes

Authors’ response:

Thanks for your suggestion.

1. We have strengthened the literature sources for 2016-2023 in the literature.

2. Our research design is rigorous, including strict design in content, structure, and method, and has certain quality standards in publishing SSCI.

3. In the article, we use the red part to indicate the modified part

2. Has the statistical analysis been performed appropriately and rigorously?

Reviewer #1: Yes

Reviewer #2: Yes

Authors’ response:

Thanks for your suggestion.

Our research data and structure have a certain quality, and it is acceptable to publish in SSCI.

3. Have the authors made all data underlying the findings in their manuscript fully available?

The [PLOS Data policy](http://www.plosone.org/static/policies.action#sharing) requires authors to make all data underlying the findings described in their manuscript fully available without restriction, with rare exception (please refer to the Data Availability Statement in the manuscript PDF file). The data should be provided as part of the manuscript or its supporting information, or deposited to a public repository. For example, in addition to summary statistics, the data points behind means, medians and variance measures should be available. If there are restrictions on publicly sharing data—e.g. participant privacy or use of data from a third party—those must be specified.

Reviewer #1: Yes

Reviewer #2: Yes

Authors’ response:

Thanks for your suggestion.

4. Is the manuscript presented in an intelligible fashion and written in standard English?

PLOS ONE does not copyedit accepted manuscripts, so the language in submitted articles must be clear, correct, and unambiguous. Any typographical or grammatical errors should be corrected at revision, so please note any specific errors here.

Reviewer #1: No

Reviewer #2: Yes

Authors’ response:

Thanks for your suggestion.

Authors’ response:

We let professional English teacher Jake to participate and help.

[www.academiccommunications.com](http://www.academiccommunications.com/).

The English is in line with the sentences and English standards published in SSCI.

5. Review Comments to the Author

Please use the space provided to explain your answers to the questions above. You may also include additional comments for the author, including concerns about dual publication, research ethics, or publication ethics. (Please upload your review as an attachment if it exceeds 20,000 characters)

Reviewer #1: This paper examines a version of the day of the week/weekend effect anomaly studies that were a common feature of the finance literature in the 1980's. Recent authors have suggested that the anomaly has largely dissipated in North America, possibly because traders take it into consideration. While the current study's results are consistent with the authors' expectations, the work is pedestrian and very basic. I'm not convinced that it would be of much interest to researchers despite the topicality of Covid-19. My main concern with the paper though is that it is barely intelligible and requires a very thorough rewriting by someone who knows the subject and whose first language is English. Beyond the exposition, the paper is constructed poorly, which, even if one struggles through the exposition, makes it difficult to understand where the authors are going. In addition, the title is unhelpful inasmuch as it gives no indication that the paper is about the "holiday effect'" in China. The motivation (why this work is important) is not well explained and inadequate attention is given to explaining why multiple indices are examined and why the results among indices differ. Finally, the construction of the Bibliography is sloppy, with incomplete references and inconsistencies in formatting.

Reviewer #2: This is a very interesting paper and my only concern for the paper is that the author(s) should give us more solid arguments about their empirical findings. What are their contributions compared to previous studies? All these need to be clarified in the revised version of paper.

Authors’ response:

Thanks for your suggestion.

Authors’ response:

We let professional English teacher Jake to participate and help.

[www.academiccommunications.com](http://www.academiccommunications.com/).

The English is in line with the sentences and English standards published in SSCI.

We have greatly improved the reading of the entire English, I believe readers can easily read the entire article.

(1) The popularity of time series only started in 1990. At that time, there was only AR, and the GARCH method had not yet
(2) GARCH was only released in 1986, GARCH+DID is completely unknown now, our research is a very innovative research method.

(3)

According to Benartzi et al. (2007), stock price movements have become a normal relationship[4]. Considering the changes in investors’ risk appetite and investment decisions during the COVID-19 pandemic, the researchers of this study raise the question: Is it due to the stock market’s volatility during the pandemic?

This type of research study is the first in mainland China, which can broaden the scope of market research in the country. The researchers of this study addressed the gaps in previous stock price research by taking into account specific factors, such as the environment and war.

The study used an innovative research method, combining GARCH and DID. Using this method, the researchers aim to strengthen the prediction correctness of the whole research.

In this study, the researchers solved the final accuracy technology for predicting stock prices, allowing investors to make more accurate decisions.

**Table.1** Illustrative research summarizing the factors that influence the share price

|  | Illustrative research | | Context | | Theoretical basis | Antecedents to share price | Key findings or propositions | |
| --- | --- | --- | --- | --- | --- | --- | --- | --- |
|  | Fama (1970) [5] | | Information share prices soon after it is discovered | | Signaling theory | Timing | The findings suggested that timing and likelihood of occurrence delivered a contribution to the  company’s residual value | |
|  | Malcolm Baker et al.(2008) [6] | | Proposed nominal stock prices catering theory | | Catering theory | Time-series | The findings revealed that there is empirical support for the predictions in both time-series and firm-level  data. | |
|  | Dolley (1933)  [7] | | Impact of nominal prices | | Trading costs depend on nominal prices theory | Timing | The findings suggested that investors refer to nominal (per-share) stock  price. | |
|  | Green and Hwang  (2007) [8] | | Linkage between stock price and low-priced stocks | | Expectation theory | Time-series | The results revealed that stocks that split experience sudden increases in comovement with low-priced stocks. | |
|  | Ohlson and Penman (1985) [9] | | Splitting stocks comove more with low-price and generally smaller-cap stocks | | Expectation theory | Time-series | The results show that stocks that split experience greater volatility increases. | |
|  | Dyl and Elliott (2006) [10] | | Cross-sectional stock price research | | Decision theory | Time-series | The findings suggested that there is a strong  cross-sectional relationship between size and share price. | |
| Black (1986) [11] | | Low price impact | | Decision theory | | Time-series | Further, investors view low-priced stocks as subject to greater noise trading. |  |

6. PLOS authors have the option to publish the peer review history of their article ([what does this mean?](https://journals.plos.org/plosone/s/editorial-and-peer-review-process#loc-peer-review-history)). If published, this will include your full peer review and any attached files.


If you choose “no”, your identity will remain anonymous but your review may still be made public.


**Do you want your identity to be public for this peer review?** For information about this choice, including consent withdrawal, please see our [Privacy Policy](https://www.plos.org/privacy-policy).

Reviewer #1: No

Reviewer #2: **Yes:** TSANG YAO CHANG

Authors’ response:

Thanks for your suggestion.
